# Effects of Psychiatric Comorbidity in Immune-Mediated Inflammatory Disease: Protocol for a Prospective Study

**DOI:** 10.2196/resprot.8794

**Published:** 2018-01-17

**Authors:** Ruth Ann Marrie, Lesley Graff, John R Walker, John D Fisk, Scott B Patten, Carol A Hitchon, Lisa M Lix, James Bolton, Jitender Sareen, Alan Katz, Lindsay I Berrigan, James J Marriott, Alexander Singer, Renée El-Gabalawy, Christine A Peschken, Ryan Zarychanski, Charles N Bernstein

**Affiliations:** ^1^ University of Manitoba Winnipeg, MB Canada; ^2^ Dalhousie University Winnipeg, MB Canada; ^3^ University of Calgary Calgary, AB Canada; ^4^ St. Francis Xavier University Antigonish, NS Canada

**Keywords:** inflammatory bowel disease, multiple sclerosis, rheumatoid arthritis, depression, anxiety, epidemiology

## Abstract

**Background:**

Immune-mediated inflammatory diseases (IMID), such as inflammatory bowel disease (IBD), multiple sclerosis (MS), and rheumatoid arthritis (RA), are highly prevalent in Canada and the United States and result in substantial personal and societal burden. The prevalence of psychiatric comorbidities, primarily depression and anxiety, in IMID exceeds those in the general population by two- to threefold, but remains underdiagnosed and undertreated. Furthermore, the effects of psychiatric comorbidity on IMID are not well understood.

**Objective:**

The objectives of this study were (1) to compare health-related quality of life and work ability in persons with IMID and psychiatric comorbidity with those of persons with IMID without psychiatric comorbidity and with those of persons with depression and anxiety disorders alone, and (2) to validate existing case identification tools for depression and anxiety in persons with IMID to facilitate improved identification of depression and anxiety by clinicians. To achieve these objectives, we designed a prospective 3-year longitudinal study. In this paper, we aim to describe the study rationale and design and the characteristics of study participants.

**Methods:**

Between November 2014 and July 2016, we recruited 982 individuals from multiple clinic and community sources; 18 were withdrawn due to protocol violations.

**Results:**

The final study sample included 247 participants with IBD, 255 with MS, 154 with RA, and 308 with depression or anxiety. The majority were white, with the proportion ranging from 85.4% (IBD [210/246]; MS [217/254]) to 74.5% (114/153, RA; *P*=.01). There was a female predominance in all groups, which was highest in the RA cohort (84.4%, 130/154) and least marked in the IBD cohort (62.7%, 155/247). Participants with depression or anxiety were more likely to be single (36.0%, 111/308) than participants in any other group (11.8% [30/255]-22.7% [56/247], *P*<.001).

**Conclusions:**

This paper presents the rationale for this study, describes study procedures, and characterizes the cohort enrolled. Ultimately, the aim is improved care for individuals affected by IMID.

## Introduction

Immune-mediated inflammatory diseases (IMID), such as inflammatory bowel disease (IBD), multiple sclerosis (MS), and rheumatoid arthritis (RA), are highly prevalent in Canada and the United States, and substantially burden affected individuals and society [1-4]. Persons with IBD, MS, and RA report poorer health-related quality of life (HRQOL) as compared with the general population [5-8]. They are also at increased risk of leaving the workforce early due to disease-related disabilities [9-11].

### Psychiatric Comorbidity

Increasingly, psychiatric comorbidity, including depression and anxiety disorders, is recognized as common among individuals with IMID, with a prevalence exceeding that in the general population by two- to threefold. Psychiatric disorders are commonly associated with adverse health outcomes, including impaired HRQOL and missed work [12,13]. The effects of psychiatric comorbidity on HRQOL and work ability in those with IMID, beyond the impact of the IMID itself, are not well understood. Most studies have focused on the impact of the IMID on cessation from paid work, but this fails to capture the spectrum of functional work impairment. Presenteeism (reduced productivity while at work due to illness) and absenteeism (missed work due to illness) have received much less attention, including how they are affected by psychiatric comorbidity [14-16]. Moreover, prior studies have often been cross-sectional and included small samples.

Several potential mechanisms may underlie the association between psychiatric morbidity and adverse outcomes. Psychiatric comorbidity may lead to changes in health behaviors such as poorer diet or sleep hygiene, lower adherence to treatment, and increased smoking [17]. Complex bidirectional relationships exist between psychiatric disorders, such as depression and anxiety, and immune function [18]. Stress may play a role in psychiatric comorbidity and poor chronic disease outcomes [19-21].

Despite the adverse effects of depression and anxiety disorders on chronic disease outcomes, these psychiatric disorders are undertreated when they co-occur in IMID [22-26]. Improved detection of these disorders in IMID is a necessary step to better management. Case identification instruments (screening tools) may promote detection of depression and anxiety disorders. However, tools developed for the general population may not translate well to use for people with IMID. For example, somatic symptoms of depression (depressive affect), such as fatigue, difficulty sleeping, and poor appetite, captured in screening tools are also common somatic symptoms of IMID, which may lead to criterion contamination [27]. Similar issues arise when screening for anxiety. Therefore, screening tools must be validated in the IMID populations. To date, these validation efforts have been limited to a few tools, and evaluation of psychometric characteristics of these tools has been limited [28-30].

### Aims

We designed a prospective 3-year longitudinal study of persons with IMID with 2 principal aims. The first aim was to compare HRQOL and work ability in persons with IMID and psychiatric comorbidity with those of persons with IMID without psychiatric comorbidity and with those of persons with depression and anxiety disorders alone. The second aim was to validate existing screening tools for depression and anxiety in persons with IMID to facilitate improved identification of depression and anxiety by clinicians. We expect that improved identification and management of these disorders would positively affect patient-centered outcomes such as HRQOL.

This paper describes the study design, recruitment of participants, and the characteristics of the established cohort.

## Methods

### Design

We enrolled participants with any of the following 5 diagnoses: (1) IBD—Crohn disease or ulcerative colitis [31,32]; (2) MS—definite diagnosis according to the Poser or revised McDonald criteria [33-36]; (3) RA—definite diagnosis based on the 2010 American College of Rheumatology/European League Against Rheumatism (ACR/EULAR) Rheumatoid Arthritis Classification Criteria [37]; (4) major depressive disorder meeting the *Diagnostic and Statistical Manual of Mental Disorders* (DSM-IV) criteria [38]; and (5) any anxiety disorder meeting the DSM-IV criteria [38]. As DSM-IV rather than DSM-5 was in place at the time of the study inception, post-traumatic stress disorder and obsessive compulsive disorder were included as anxiety disorders, in keeping with the DSM-IV classification scheme.

All participants were required to be aged 18 years or older (without an upper age limit), able to provide informed consent, willing to participate in the study for 3 years, and to have an adequate knowledge of the English language to complete questionnaires (this latter criterion did not exclude anyone). Diagnoses of IBD, MS, and RA were confirmed by medical records review and by querying treating physicians directly if needed. Diagnoses of depression or anxiety were confirmed by structured clinical interview as delineated below. The presence of comorbid psychiatric disorders was not an exclusion criterion.

We obtained ethics approvals from the University of Manitoba Health Research Ethics Board and research committee approval from the Winnipeg Regional Health Authority, Winnipeg Health Sciences Centre, St. Boniface Hospital, Seven Oaks Hospital, and Victoria General Hospital. All participants provided written informed consent. In addition to consenting to core study activities, participants were also asked to agree to blood sample collection at each visit and to linkage of the collected data to health administrative data records.

### Recruitment

We used general and targeted strategies to recruit participants from the community and tertiary care settings. General strategies included the placement of posters in local hospitals, private medical and psychology clinics, and academic institutions within the Winnipeg region; use of social media outlets (tweets and Facebook posts) through the largest tertiary center (Winnipeg Health Sciences Centre); and self-help groups for mental health concerns. The targeted strategies used for each group are described below.

For IBD, research assistants directly approached individuals attending gastroenterology clinic visits using a standardized script and contacted participants in a population-based IBD research registry via email. The registry was established in 1995 by one of the investigators (CB) and includes nearly half the provincial IBD population [39].

For MS, research assistants contacted participants in the Winnipeg MS Clinic registry by phone and mail. This clinic was established in 1998 and is the sole source of care for Manitobans with MS being treated with disease-specific therapies, although the clinic population is not limited to those receiving such therapies. A research registry was established in 2011, and most of the individuals with MS attending the clinic have agreed to participate.

For RA, research assistants directly approached individuals attending scheduled clinic visits at the University of Manitoba Arthritis Centre, the tertiary care clinic for rheumatologic disease in Manitoba, using a standardized script. The University of Manitoba Arthritis Centre also contacted individuals with RA in their clinic database by mail, as did one other local community rheumatology clinic.

For depression and anxiety disorders, information about the study was included in appointment letters sent to individuals referred for psychology or psychiatry consultation at tertiary care centers within the Winnipeg region. Information letters were also mailed by primary care clinics operated in partnership by the Winnipeg Regional Health Authority and the University of Manitoba and at a local primary care practice to patients with diagnoses of depression or an anxiety disorder identified using their electronic medical records. Research coordinators also presented information about the study to individuals attending psychoeducational classes for cognitive behavioral therapy held by the Psychiatry Program at the Health Sciences Centre.

### Data Collection and Measures

We collected information regarding sociodemographic characteristics, height, weight, blood pressure, physical function, cognitive function, psychiatric morbidity, and self-reported smoking status, stress, pain, fatigue, HRQOL, and work disability from all participants. Disease-specific measures tailored to each disease group were also collected. Unless otherwise specified, each of these measures was collected at study enrollment and will be collected at 3 annual study-specific assessments thereafter for a total of 4 assessments. Annual assessments will be booked within ±1 month of the enrollment date.

#### Sociodemographic Characteristics

Using questionnaires, participants reported sex, date of birth, ethnicity, total number of years of formal education, highest level of education attained, annual household income, marital status, whether they had any children (yes vs no), and current or most recent occupation. Ethnicity was captured using the categories specified by Statistics Canada in regular surveys. Highest level of education completed was reported using the following categories: elementary school, junior high school, high school diploma or General Education Diploma (GED), college, technical/trade, university bachelor’s degree, university master’s degree, university doctorate, and other. Annual household income was reported as less than Can $15,000, Can $15,000-29,999, Can $30,000-49,999, Can $50,000-100,000, more than Can $100,000 or “I do not wish to answer.” Responses for marital status included single/never married, married, common law, divorced, widowed, and separated. Occupation was categorized using Statistics Canada’s categories as management; business, finance, and administration occupations; health occupations; occupations in education, law, and social, community, and government services; occupations in art, culture, recreation, and sport; sales and service occupations; trades, transport and equipment operators, and related occupations; natural resources, agriculture, and related production occupations; and occupations in manufacturing and utilities [40].

#### Body Mass Index

Research assistants measured height and weight to derive body mass index (kg/m^2^).

#### Blood Pressure

Blood pressure and heart rate were measured in the seated position using an automatic blood pressure machine.

#### Physical Function

We assessed lower limb function and ambulation using the timed 25-foot walk test [41-43]. This tool is considered to be a quick, reliable measure of functional capacity in older populations [44,45] and has been validated for use in MS. Upper limb function was assessed using the 9-hole peg test, a validated measure used in MS [41-43] and the Arthritis Hand Function Test for RA [46]. The timed 25-foot walk test and 9-hole peg test constitute 2 of the 3 components of the Multiple Sclerosis Functional Composite [41-43], which all research assistants were certified to perform.

#### Cognitive Function

We selected validated neuropsychological measures to assess cognitive domains of processing speed, working memory, and verbal learning, which are often found to be affected in MS [47], RA [48], and IBD [49] as well as in depression (processing speed, working memory) and generalized anxiety disorder [50]. The measures included the Symbol Digit Modalities Test (processing speed) [51]; Wechsler Memory Scale-III Letter-Number Sequencing subtest (working memory) [52]; and California Verbal Learning Test II (verbal learning and memory) [53]. We also included the Wechsler Test of Adult Reading as a measure of premorbid intellectual functioning [54]. The extent of cognitive testing was constrained by the length of study visits and the potential for participant fatigue. Research assistants who administered the cognitive measures were trained by a psychometrician, under the supervision of a registered neuropsychologist (JDF).

#### Smoking Status

We assessed smoking status because smoking may confound associations between psychiatric status and study outcomes, such as HRQOL [55-57]. Individuals who reported ever smoking ≥100 cigarettes were defined as ever smokers [58] and were asked to report current smoking status (not at all, some days, every day), the age at which they had started smoking, how many cigarettes they currently smoked per day, and the number of days in the past 30 days that they had smoked. Ex-smokers reported the age at which they quit smoking cigarettes and the average number of cigarettes smoked per day during the years that they smoked.

#### Comorbidity

To assess self-reported physical and psychiatric comorbidities, we used questions derived from a survey validated for the general population [59] and for use for people with MS [60]. Participants reported whether a doctor has ever diagnosed them with any of the following conditions: high cholesterol, high blood pressure, heart trouble, disease of arteries in the legs, lung trouble, diabetes mellitus, cancer of the breast, cancer of the colon or rectum, cancer of the lung, skin cancer, other cancers, migraine, thyroid disease, lupus, osteoarthritis, osteoporosis, fibromyalgia, kidney disease, peptic ulcer disease, liver problems, irritable bowel syndrome, epilepsy (seizure disorder), depression, anxiety disorder, bipolar disorder, or schizophrenia. For any condition indicated as present, participants reported the year of diagnosis and whether it is currently being treated.

#### Psychiatric Morbidity

We assessed psychiatric morbidity at enrollment using the structured clinical interview for DSM-IV-TR Axis I Disorders—Research version (SCID), a semistructured interview to identify anxiety, mood, and substance use disorder DSM-IV diagnoses [61], all of which were captured in this study. The interviews were conducted by graduate students in clinical psychology, nurses, and research coordinators who were trained to conduct the interviews by a registered clinical health psychologist (JRW) with extensive experience with SCID. Training included review of the SCID users guide, observing video examples of interviews, detailed review of the modules, role-playing interviews, observing an interview, and being observed when administering an interview. Team members met to review interviews regularly, and consultation with more experienced team members was available when diagnostic questions were encountered.

Participants also completed several case identification (screening) instruments for depression and anxiety (Table 1) [62-68]. These instruments were selected because they were brief, easy to self-administer, and available in the public domain for clinical purposes, making them feasible to use in clinical settings. In the general population, they share features of moderate to high sensitivity and specificity [62-68]. In addition, the “Perceived Need Question” was included, which queries patients’ perceived need for treatment and can improve specificity of depression screening. Responses are “no,” “yes, but not today,” or “yes” [69].

#### Stress

The Perceived Stress Scale (PSS) is widely used to measure the degree to which individuals are experiencing stress, the underlying concept being that stress is the extent to which perceived demands exceed the perceived personal resources to cope [70]. The 10-item version (PSS-10) has high internal consistency reliability and test-retest reliability, good construct validity, and predictive validity [71-74]. Scores range from 0 to 40, with higher scores indicating greater perceived stress.

#### Pain

The Pain Effects Scale assesses pain, and it was originally developed and validated for the Medical Outcomes Study [75]. A reduced 6-item version (Modified Pain Effects Scale) was included in the MS quality of life inventory, and is valid and reliable [76,77]. Scores range from 6 to 30, with higher scores indicating greater pain.

#### Fatigue

Fatigue was evaluated using the Fatigue Impact Scale for Daily Use (D-FIS), a brief validated instrument adapted from the Fatigue Impact Scale that includes 8 items that reflect daily fatigue [78]. Each item is scored ordinally from 0 (no problem) to 4 (extreme problem), and total scores range from 0 to 32. The D-FIS has good psychometric properties [79].

#### Health-Related Quality of Life

We measured HRQOL using the Short Form-36, a generic measure of HRQOL validated in the general population as well as in multiple IMID populations [80-83]. The 2 summary scales capture physical HRQOL (the physical component score [PCS]-36) and mental HRQOL (the mental component score [MCS]-36). Scores on the PCS-36 and MCS-36 range from 0 to 100, with a mean of 50 and standard deviation of 10; higher scores indicate better quality of life.

#### Work Impairment

We used the Work Productivity and Activity Impairment Questionnaire (WPAI), a 6-item questionnaire to measure work and activity impairment. Impairment due to a specified health problem during the past 7 days is reported, where higher scores indicate greater impact of health. Outcomes include percentage of work time missed (absenteeism), percentage of impairment while working (presenteeism), percentage of overall work impairment, and percentage of activity impairment due to health problems. The first 3 outcomes are calculated for persons who are working for pay, and the last outcome is calculated for all persons. In a clinical trial for IBD, the WPAI had adequate discriminative validity, reliability, and responsiveness [84]; it has good construct validity in RA [14], and it has been used in MS studies [15].

#### Disease-Specific Measures

The disease-specific measures were chosen to describe disease activity and disease progression or functional status.

**Table 1:** Case identification (screening) instruments for depression and anxiety.

Screening instruments for depression and anxiety	Construct	Number of items	Scoring	Published cut points	Possible range of values
Patient Health Questionnaire—brief (PHQ-2) [63] (based on the first 2 questions in this scale)	Depression (presence of)	2	Items scored 0-3, summed	3	0-6
Patient Health Questionnaire (PHQ-9) [62]	Depression (presence of)	9	Items scored 0-3, summed	10	0-27
Generalized Anxiety Disorder 7-Item Scale [64]	Anxiety, generalized (severity of)	7	Items scored 0-5, summed	10	0-21
Overall Anxiety and Severity Impairment Scale [65]	Anxiety (severity of)	5	Items scored 0-4, summed	8	0-20
Hospital Anxiety and Depression Scale [66]	Depression (severity of), anxiety (severity of)	14 (7 for anxiety, 7 for depression)	Items scored 0-3, summed	8, 11	0-21
Kessler-6 Distress Scale [67]	Nonspecific distress	6	Items scored 1-5, summed	19	6-30 (alternative scoring)
National Institutes of Health Patient-Reported Outcomes Measurement Information System—emotional distress depression—Short Form 8a [68]	Depression (severity of)	8	Items scored, summed, and then converted to T score	T-score 60	8-40, T score 38.2-81.3
National Institutes of Health Patient-Reported Outcomes Measurement Information System—emotional distress anxiety—Short Form 8a [68]	Anxiety (severity of)	8	Items scored, summed, and then converted to T score	T score 60	8-40, T score 37.1-83.1

##### Disease Activity

We characterized disease activity using the Powell Tuck Index (PTI) for ulcerative colitis [85], the Harvey Bradshaw Disease Activity Index (HBDAI) for Crohn disease [86], annualized relapse rate for MS, and Clinical Disease Activity Index (CDAI) [87] for RA. The PTI and the HBDAI inquire about symptoms over the previous week and are administered by research staff. The HBDAI includes a score for the presence or absence of an abdominal mass as assessed by an abdominal exam conducted by trained personnel. A score of ≥5 is considered active disease on each index. The CDAI is a composite obtained by summing 28 tender and 28 swollen joint counts and disease activity according to the patient (0-10) and the physician (0-10) [88]. It has the advantage of not requiring any laboratory tests. A score of 0-2.8 indicates remission, 2.9-10.0 indicates low activity, 10.1-22.0 indicates moderate activity, and 22.1-76.0 indicates high activity [89].

##### Disease Progression

Participants reported the year of symptom onset and the month and year of diagnosis of their IMID. Dates of symptom onset and diagnosis were verified using medical records. Current disease-modifying therapies were also captured from medical records. We characterized disease phenotype and progression in IBD using the Montreal Classification [31,90], which identifies 4 key variables in Crohn disease including age of onset, disease behavior (inflammatory, stricturing, or fistulizing disease), disease location (ileal only, colon only, small bowel and colon, and upper gastrointestinal tract), and whether perineal fistulas are present or not. In ulcerative colitis, the Montreal Classification identifies age of onset and extent of disease (either rectal, left-sided, or pancolitis). For MS, we used the Expanded Disability Status Scale (EDSS) [91], which is an ordinal measure of disability based on the neurological examination. Total EDSS scores range from 0 (no disability) to 10 (death due to MS), and are derived from scores on visual, brainstem, pyramidal, sensory, cerebellar, sphincter, and cerebral functional systems, as well as an observed walk of up to 500 meters. In RA, we used the modified Health Assessment Questionnaire (mHAQ) [92,93]. The mHAQ is a patient-reported measure that assesses functional status, specifically the degree of difficulty (without difficulty, 0; with some difficulty, 1; with much difficulty, 2; unable to do, 3) to perform 8 daily activities (dressing and grooming, arising, eating, walking, hygiene, reach, grip, and common daily activities). Responses are averaged to yield scores ranging from 0 to 3. Where available, x-rays obtained at annual clinic visits will be used to determine the presence of joint erosions.

### Sample Collection

At the end of each assessment, consenting participants provided a blood sample collected by venipuncture using a straight needle into an EDTA tube and into a Paxgene (Qiagen) deoxyribonucleic acid (DNA) tube. Samples collected in EDTA tubes were centrifuged at 1500-2500 × g for 15 min at room temperature. The resulting plasma layer and buffy coat layers were aliquoted separately into 2 mL cyro vial tubes for storage at −80°C. DNA was extracted using a Paxgene DNA kit using a single tube procedure according to the manufacturer’s (Qiagen) instructions and stored at −80°C.

### Data Management

Study data were managed using REDCap electronic data capture tools hosted at the University of Manitoba [94]. REDCap (Research Electronic Data Capture) is a secure, Web-based application designed to support data capture for research studies, providing an intuitive interface for validated data entry, audit trails for tracking data manipulation and export procedures, automated export procedures for seamless data downloads to common statistical packages, and procedures for importing data from external sources [94].

### Sample Size

The required sample size for the 2 principal aims was determined as follows. For the first aim, to test the baseline association between psychiatric comorbidity and either HRQOL or work disability in each IMID group, we assumed that at least 30% of the sample will experience any psychiatric comorbidity [95-97], confounders will explain 10% of the variation in the data, a 10% difference in HRQOL (MCS-36/PCS-36), a 10-point difference in work impairment for the 2 psychiatric disorder (depression/anxiety) groups, a pooled standard deviation of 10, and alpha=.05. For longitudinal analyses, we assumed an annual attrition rate of 3% [98], an average annual rate of decline of 3% in HRQOL and work impairment, a pooled between-group variance of 10, and a pooled within-group variance of 5. On the basis of these assumptions, each IMID and psychiatric group would need a minimum sample size of 150, for a total of 750.

For the second aim, which sought to test the performance of the case identification (screening) tools for depression and anxiety as compared with the SCID in the IMID groups, we assumed a lower bounds sensitivity=0.75 and specificity≥0.85 [62,99], precision=0.15, and alpha=.05, for which the required sample size per group was 247. To detect a kappa (κ) for agreement ≥0.60 between the SCID and the screening tools, assuming alpha=.05, beta=.20, a null hypothesis of kappa≥.46, and that depression or anxiety affects ≥15% of the IMID cohort, the required sample size per group was 250.

Therefore, we sought to recruit 250 participants for each IMID group, and 150 each for the depression and anxiety disorder groups.

### Current Analysis

We summarized the characteristics of the sample at enrollment for the purpose of assessing the potential generalizability of the findings. We summarized categorical variables using frequency (percentage) and continuous variables using mean (standard deviation) or median (25th-75th percentiles). Each of the disease groups was also reviewed relative to samples in other studies of those disease groups, to consider representativeness and generalizability of the samples. Statistical analyses were conducted using SAS V9.4 (SAS Institute Inc., Cary, NC).

### Future Analyses

We will assess the impact of changes in psychiatric status on change in these HRQOL and work ability outcomes over the 4 measurement occasions (3-year period) using generalized linear models with generalized estimating equations to account for the dependence among the repeated measurements. We will select the distribution to model each outcome using a combination of empirical (eg, ratio of model deviation to its degrees of freedom) and theoretical considerations. We will choose a correlation structure for the repeated measurements by examining the pattern of empirical correlations. The independent variables of interest will be IMID type and psychiatric status. Psychiatric status will be determined based on the SCID-determined diagnoses (at enrollment) and symptom severity based on whichever screening instrument has the best performance characteristics (as described below). Potential confounding covariates will be age, sex, disease duration, education, smoking, body mass index, physical comorbidity, and disease activity status.

On the basis of published cut points, we will compare depression and anxiety status based on the SCID and the screening instruments using sensitivity, specificity, positive predictive value, and negative predictive value. We will also use receiver operating curve analysis to understand the relationship between sensitivity and the false positive rate, which allows an optimal cut point to be assigned depending on the requirements in a specific context. We will assess internal consistency reliability using Cronbach alpha [100]. In a subgroup of ~150 participants in each IMID group, we will determine the test-retest reliability of these instruments using an intraclass correlation coefficient.

## Results

### Recruitment

Between November 2014 and July 2016, we enrolled 982 individuals. Of these, 18 were later withdrawn; 6 did not meet inclusion criteria after review of their medical records because they did not have confirmed diagnoses of IBD (3) or RA (2), or had 2 of the IMID of interest (1). A total of 11 individuals enrolled as members of the psychiatric cohort were withdrawn as they did not have a confirmed diagnosis of depression or anxiety disorder following SCID administration. Finally, 1 individual in the IBD cohort withdrew from the study and that person’s data were destroyed shortly after enrollment. Therefore, the final study population included 247 participants with IBD, 255 with MS, 154 with RA, and 308 with depression or anxiety (172 with depression, 136 with an anxiety disorder as self-identified at enrollment) for a total of 964. As expected, participants were recruited from multiple sources (Table 2). A higher proportion of participants with MS were recruited through targeted contacts, reflecting the delivery of MS care in Manitoba through a single specialty clinic, whereas care for other conditions is less centralized.

**Table 2:** Recruitment sources for study participants.

Source	Inflammatory bowel disease (N=247), n (%)	Multiple sclerosis (N=255), n (%)	Rheumatoid arthritis (N=154), n (%)	Depression/anxiety disorder (N=308), n (%)
Targeted email/mail^a^	98 (39.7)	222 (87.0)	52 (33.8)	59 (19.1)
Clinic/CBT class^b^	133 (53.8)	30 (11.8)	80 (51.9)	60 (19.4)
Poster (paper/electronic)	5 (2.0)	3 (1.2)	10 (6.5)	60 (19.4)
Tweets/Facebook/Internet	5 (2.0)	0 (0)	0 (0)	15 (4.9)
News article	1 (0.4)	0 (0)	0 (0)	2 (0.6)
Clinician referral	0 (0)	0 (0)	0 (0)	7 (2.3)
Word of mouth	1 (0.4)	0 (0)	12 (7.8)	18 (5.8)
Unknown	4 (1.6)	0 (0)	0 (0)	87 (29.2)

^a^Some participants in the MS Clinic registry were contacted by telephone rather than by mail.

^b^Cognitive behavioral therapy (CBT) classes served as a recruitment source only for those with depression/anxiety.

Sociodemographic characteristics of the participants are shown in Table 3. Several differences were apparent across disease groups. The percentage of participants who were white varied across groups, being highest among those with IBD or MS and lowest among those with RA. We observed a female predominance in all groups, but this was least marked in the IBD cohort. Participants with depression or anxiety were more likely to be single than participants in any other group. Annual income also varied across groups, being highest in the IBD cohort.

### Individual Immune-Mediated Inflammatory Diseases Cohorts

As has been observed in other IBD cohorts, participants were more likely to be females than males (Multimedia Appendix 1) [101-103]. The proportion of individuals reporting white background was higher in this study than that of a national US study [101], but lower than that seen in a previous Manitoba study [102]. The level of education was generally high, consistent with other studies [101-103]. Participants in the present MS cohort were older than those in 3 other Canadian MS study cohorts [104,105], and a slightly higher proportion of females was observed also (Multimedia Appendix 2). The age at MS onset was slightly lower in this study than in the other cohorts. The proportion of female participants in the present RA cohort was higher than that reported in other RA cohorts, and the proportion with white ethnicity was lower (Multimedia Appendix 3) [106-108]. The second most common ethnicity in our cohort was First Nations and other Indigenous ethnicities (not specified).

The proportion of women in the depressed/anxiety disorder cohort was higher than that in a previous cohort from the Canadian Community Health Survey—Mental Health (CCHS) in 2012 (Multimedia Appendix 4). The percentage with more than a high school education level was similar in both cohorts. The proportion who smoked was lower in our cohort than in the CCHS cohort.

**Table 3:** Cohort demographics stratified by disease group.

Characteristics		Total (N=964)	Inflammatory bowel disease (N=247)	Multiple sclerosis (N=255)	Rheumatoid arthritis (N=154)	Depression/anxiety disorder (N=308)	*P* value
Age in years, mean (SD^a^)		49.2 (14.2)	47.4 (14.8)	51.1 (12.9)	59.5 (11.7)	43.9 (13.0)	
**Sex, n (%)**							<.001
	Male	235 (24.4)	92 (37.2)	47 (18.4)	24 (15.6)	72 (23.5)	
	Female	729 (75.6)	155 (62.7)	208 (81.6)	130 (84.4)	235 (76.6)	
**Ethnicity, n (%)**							
	White	786 (81.9)	210 (85.4)	217 (85.4)	114 (74.5)	245 (79.8)	.01
	Other	174 (18.1)	36 (14.6)	37 (14.5)	39 (25.5)	62 (20.2)	
	Missing	4	1	1	1	1	
**Education, n (%)**							.06
	Elementary school	5 (0.5)	0 (0)	1 (0.4)	3 (1.9)	1 (0.3)	
	Middle school	44 (4.5)	8 (3.2)	9 (3.5)	11 (7.1)	16 (5.2)	
	High school or GED^b^	268 (27.8)	68 (27.5)	78 (30.6)	37 (24.0)	85 (27.6)	
	College	253 (26.2)	51 (20.6)	72 (28.2)	45 (29.2)	85 (27.6)	
	Technical or trade	107 (11.1)	32 (13.0)	29 (11.4)	19 (12.3)	27 (8.8)	
	Bachelor’s degree	215 (22.3)	60 (24.3)	56 (22.0)	27 (17.5)	72 (23.4)	
	Master’s degree	54 (5.6)	21 (8.5)	7 (2.8)	10 (6.5)	16 (5.2)	
	Doctoral degree	18 (1.9)	7 (2.8)	3 (1.2)	2 (1.3)	6 (1.9)	
**Annual income, n (%)**							<.001
	Less than Can $15,000	100 (10.4)	14 (5.7)	20 (7.8)	19 (12.3)	47 (15.3)	
	Can $15,000-29,999	88 (9.1)	14 (5.7)	24 (9.4)	19 (12.3)	31 (10.1)	
	Can $30,000-49,999	162 (16.8)	29 (11.7)	37 (14.5)	32 (20.8)	64 (20.8)	
	Can $50,000-100,000	341 (35.4)	104 (42.1)	99 (38.8)	48 (31.2)	90 (29.2)	
	More than Can $100,000	189 (19.6)	68 (27.5)	47 (18.4)	25 (16.2)	49 (15.9)	
	I do not wish to answer	84 (8.7)	18 (7.3)	28 (11.0)	11 (7.1)	27 (8.8)	
**Marital status, n (%)**							<.001
	Single or never married	217 (22.5)	56 (22.7)	30 (11.8)	20 (13.0)	111 (36.0)	
	Married or common law	569 (59.0)	160 (64.8)	182 (71.4)	93 (60.4)	134 (43.5)	
	Divorced or separated	150 (15.6)	24 (9.7)	39 (15.3)	31 (20.1)	56 (18.2)	
	Widowed	28 (2.9)	7 (2.8)	4 (1.6)	10 (6.5)	7 (2.3)	

^a^SD: standard deviation.

^b^GED: General Education Diploma.

## Discussion

This paper presents the study rationale, describes study procedures, and characterizes the cohort enrolled. The ultimate goal of the study was to compare and contrast the impact of psychiatric comorbidity on outcomes in IMID that affect different organ systems but share the issues of immune dysregulation and inflammation. We hope to gain more specific insights into the role of psychiatric comorbidity in IMID, with the aim of improved care for individuals affected by IMID. These analyses will be conducted once the cohort completes follow-up.

When selecting the measures for this study, we sought standardized measures appropriate for the domains of interest. Where possible, we chose instruments with good criterion and construct validity, and high reliability, which had been demonstrated in one or more of the disease groups of interest. If this was not possible, we favored validated measures used in Canadian national data collection efforts to offer the opportunity for comparisons of this cohort with national cohorts.

### Participants

Our recruitment strategy precluded the determination of a participation rate, as it was designed to reach potential participants from a broad range of settings using general and targeted strategies. For all of the groups except for the RA group, we were able to recruit the desired number of participants; the time period for recruitment was limited by the need to complete 3 years of follow-up by the end of the funding period. It has been noted that participation rates in cohort studies and surveys have declined over the last 3 decades [109]. Several factors have been suggested to contribute to this decline [109]. These include an increasing array of research and marketing studies, a decrease in volunteerism, and the complexity of study demands. The latter is likely to be a particular issue in our study given the length of study visits, which range from 1.5 to 3 hours depending on the disease group, and the requested duration of participation (3 years). Nonetheless, our sample size remains large enough to meet the study’s main objectives.

Retention of study participants will be critical, and we aimed to minimize attribution by employing several strategies that have been successful in other studies [110,111]. First, participants are offered gift cards to recognize their contributions to the study at each study visit as this has been shown to improve response rates [112]. Second, retention is carefully tracked. All participants receive reminder phone calls or emails for study appointments depending on their preference. Study coordinators are flexible about rescheduling appointments and follow-up with all participants who miss appointments. Third, a study newsletter is distributed semiannually. The newsletter provides information about study team members, progress, and findings, as well as information about relevant research news and wellness. We hope that annual face-to-face contact will also aid retention. Finally, when participants move out of province or become too ill to attend study visits, they are offered the opportunity to maintain participation by completing some of the study assessments such as questionnaires by telephone or mail.

The characteristics of participants enrolled in our study are similar to those enrolled in other studies after accounting for differences in how variables were measured, supporting the representativeness of the sample across the disease groups. However, some differences with respect to other cohorts and the general IMID populations of interest are worth noting. Participants in the MS cohort were older than those in some other cohorts, likely reflecting recruitment efforts aimed at capturing the full spectrum of individuals with MS. The proportion of participants with MS who were women was slightly higher than expected, even when considering that women are affected by MS two to three times more often than men [113]. Similarly, the proportion of individuals with RA or depression/anxiety disorder who were women was also somewhat higher than expected. The proportion of individuals reporting white race/ethnicity was lower in our RA cohort than that observed in other cohorts, but this may better reflect the local demographics, as there is a large First Nations (Indigenous) population in Manitoba [114], and there is a high risk of RA in this group. There was a very high proportion of participants with RA who were actively being treated with disease-modifying antirheumatic drugs, potentially reflecting oversampling of participants from a tertiary care center relative to community sampling. The level of education in all recruited cohorts was relatively high.

### Limitations

Selection bias is a potential limitation of any study. As reviewed elsewhere [109], certain demographic characteristics are associated with participation in research. Women are more likely to participate in research studies than men. Findings regarding age are inconsistent, as are findings regarding race/ethnicity. Individuals with higher levels of education and higher socioeconomic status are more likely to participate. However, in studies focused on a particular health condition or exposure, health status and relevance of the study subject to the potential participant also influence participation and may modify these demographic patterns [109].

In addition to potential selection biases, attrition may occur despite the use of appropriate retention strategies due to death, loss to follow-up, or other reasons. As noted, we did not achieve the desired sample size for the RA cohort, and this will reduce statistical power for RA-specific analyses. To minimize participant burden, we included only a single measure of pain and of fatigue, although these are multidimensional concepts that may be better evaluated with multiple instruments that capture different dimensions. We did not capture all potential confounders such as the life events (eg, pregnancy, menopause) that may be unique to subgroups of the study population and that may be associated with disease activity and psychiatric status in IMID [115-117].

### Conclusions

Nonetheless, this study has several strengths including the establishment of representative cohorts of those with IMID and depression and anxiety disorders, which will support comparative work, as well as careful attention to retention and stakeholder engagement. We expect this comprehensive prospective longitudinal study to provide valuable new knowledge about the impact of psychiatric comorbidity on IMID, including on outcomes important to affected individuals and society, such as HRQOL and work disability. We also expect that this study will contribute to improved diagnosis of psychiatric comorbidity by identifying validated instruments which can be used in clinical practice. For health care providers, an understanding of the relationships between psychiatric disorders, symptoms of pain, and fatigue and outcomes should encourage more attention to the identification and treatment of psychiatric disorders and change the approach to disease management to improve outcomes in IMID. The analytical methods used will support future research regarding patient-reported outcome measures. Focusing on three IMID with similarities and differences will support the generalizability of our findings to other IMID and provide policy makers with the evidence base to make decisions regarding health services for IMID broadly.

## Appendix

Multimedia Appendix 1 Characteristics of participants with inflammatory bowel disease (IBD) and those of participants in other IBD cohorts.

Multimedia Appendix 2 Characteristics of participants with multiple sclerosis (MS) and those of participants in other Canadian MS studies.

Multimedia Appendix 3 Characteristics of participants with rheumatoid arthritis (RA) and those of participants in other RA cohorts.

Multimedia Appendix 4 Characteristics of participants with depression or anxiety disorder and those of participants in other depression or anxiety disorder cohorts.

## Abbreviations

ACR/EULAR: American College of Rheumatology/European League Against Rheumatism
CCHS: Canadian Community Health Survey
CDAI: Clinical Disease Activity Index
D-FIS: Fatigue Impact Scale for Daily Use
DNA: deoxyribonucleic acid
EDSS: Expanded Disability Status Scale
GED: General Education Diploma
HBDAI: Harvey Bradshaw Disease Activity Index
HRQOL: health-related quality of life
IBD: inflammatory bowel disease
IMID: immune-mediated inflammatory diseases
MCS: mental component score
mHAQ: Modified Health Assessment Questionnaire
MS: multiple sclerosis
PCS: physical component score
PSS: Perceived Stress Scale
PTI: Powell Tuck Index
RA: rheumatoid arthritis
SCID: structured clinical interview for DSM-IV-TR Axis I Disorders—Research version
SD: standard deviation
WPAI: Work Productivity and Activity Impairment Questionnaire
