# Supplementary material for: Effects of Psychiatric Comorbidity in Immune-Mediated Inflammatory Disease: Protocol for a Prospective Study
Source: JMIR Res Protoc. 2018 Jan 17;7(1):e15. doi: 10.2196/resprot.8794 (PMC5792704; doi:10.2196/resprot.8794)
Supplement: Multimedia Appendix 1 [file resprot_v7i1e15_app1.pdf]

Multimedia Appendix 1. Characteristics of participants with inflammatory bowel disease (IBD) and those of participants in other IBD cohorts

**Characteristic**	**Present study**	**Bhandari[88]**	**Ediger[89]**	**De Groof[90]**
Data collection years	2014-2016	2009-2010	2003-2004	2004-2012
Study name	-	NHANES[c]	Manitoba IBD Cohort	-
Region	Manitoba, Canada	United States	Manitoba, Canada	Netherlands
N	247	2325226[d]	304	1461
**Age, yr** mean (SD)	47.4 (14.8)		41 (14.06)	
**Age, yr,** n (%)				
20-50	122 (49.4)	50%	e	
>50	118 (47.8)	50%	78 (25.6)	
**Sex**, n (%)				
Male	92 (37.2)	42%	121 (39.8)	595 (40.7)
Female	155 (62.7)	58%	183 (60.2)	866 (59.3)
**Ethnicity**, n (%)				
Caucasian	210 (85.4)	81%	289 (95.1)	
Other	36 (14.6)	19%	15 (4.9)	
Missing	1	0	0	
**Education**, n (%)				
<High school	8 (3.2)	21%		
High School/ GED	68 (27.5)	7%		
Some College	78 (31.6)	40%		
≥College/university	93 (37.6)	32%	76 (25.0)	
**Marital Status**, n (%)				
Single/never married	56 (22.7)	19%	-	

Married/common law	160 (64.8)	56%	201 (66.1)	
Divorced/separated/widowed	31 (12.5)	25%	-	
**Ever smoked**	138 (55.9)	51%		
**Current smoker**	186 (19.3)	-		
**Overweight/obese**	151 (61.1)	57%		
**Number of comorbid conditions**[a]				
0	138 (55.9)	29%		
1	73 (29.5)	44%		
2	26 (10.5)	21%		
≥3	10 (4.0)	6%		
Age at IBD onset, yrs mean (SD)	26.6 (13.2)[b]			-
Age at IBD diagnosis, yrs mean (SD)	30.4 (13.4)[b]			35.0 (25-48)
**Type of IBD**				
Crohn's disease	153 (61.9)		165 (54.2)	761 (52.1)
Ulcerative colitis	94 (38.1)		143 (47.0)	579 (39.5)
Unspecified	0 (0)		0 (0)	121 (8.3)
**Montreal classification UC**				
Extent[c]				
E1	14 (14.9)			178/749 (23.8)
E2	37 (39.4)			345/749 (46.1)
E3	39 (41.5)			226/749 (30.2)
**Montreal classification CD**				
Age at diagnosis[d]				
A1	20 (13.2)			35/571 (6.1)
A2	101 (66.9)			370/571 (64.8)
A3	30 (19.9)			166/571 (29.1)
Localization				
L1	65 (42.5)			172/565 (30.4)
L2	21 (13.7)			182/565 (32.2)

L3	67 (43.8)	205/565 (36.3)
L4	5 (3.3)	6/565 (1.1)
Behaviour		
B1	63 (41.2)	299/555 (53.9)
B2	57 (37.2)	119/555 (21.4)
B3	40 (26.1)	137/555 (24.7)

a- Self-reported comorbidities in present study: hypertension, diabetes, heart disease (including coronary artery disease, congestive heart failure), liver disease, kidney disease, cancer (breast, lung, colon, skin), chronic lung disease (including asthma, chronic obstructive pulmonary disease). Self-reported comorbidities in NHANES study: hypertension, diabetes, CAD, stroke, CHF, liver disease, kidney disease, asthma, cancer. b- 2 missing age at onset and 3 missing age at diagnosis. c- NHANES = National Health and Nutrition Examination Survey; sample characteristics reported only as percentages. d-NHANES using a multistage, probability sampling design to derive estimates; e-study included individuals under age 20 years, so comparable information not reported
