# Supplementary material for: Effects of Psychiatric Comorbidity in Immune-Mediated Inflammatory Disease: Protocol for a Prospective Study
Source: JMIR Res Protoc. 2018 Jan 17;7(1):e15. doi: 10.2196/resprot.8794 (PMC5792704; doi:10.2196/resprot.8794)
Supplement: Multimedia Appendix 2 [file resprot_v7i1e15_app2.pdf]

Multimedia Appendix 2. Characteristics of participants with multiple sclerosis (MS) and those of participants in other Canadian MS studies

**Characteristic**	**Present study**	**Fiest[91]**	**Metz[92]**	**Metz [92]**
Data collection years	2014-2016	2010-2013	2011-2013	2002-2006
Study name	-	Epidemiology and impact of comorbidity in MS	The Alberta MS Initiative (TAMSI)	Calgary Research Cohort
Region		Multi-site, Canada	Alberta, Canada	Calgary, Canada
N	255	949	704	1717
**Mean age** (SD)	51.1 (12.9)	48.6 (11.4)	47.6 (11.5)	45.9 (11.1)
**Sex**, n (%)				
Male	47 (18.4)	235 (24.8)	159 (22.6)	393 (22.9)
Female	208 (81.6)	714 (75.2)	545 (77.4)	1324 (77.1)
**Ethnicity**, n (%)				
Caucasian	217 (85.4)	810 (85.4)	685 (95.6)	1613 (95.1)
Other	37 (14.5)	139 (14.6)	19 (4.4)	104 (4.9)
**Education**, n (%)				
<High school	10 (3.9)		32 (4.6)	154 (9.1)
High School/ GED	78 (30.6)	258 (30.1)[a]	170 (24.4)	479 (28.3)
College/Technical/Trade	101 (39.6)	574 (67.1)[b]	268 (38.5)	675 (39.8)
Bachelor’s degree	56 (22.0)		163 (23.4)	288 (17.0)
>Bachelor’s degree	10 (4.0)		64 (9.2)	99 (5.8)
**Marital Status**, n (%)				

Single/never married	30 (11.8)		88 (12.6)	182 (10.6)
Married/common law	182 (71.4)		519 (74.0)	1267 (74.1)
Divorced/separated/widowed	43 (16.9)		94 (13.4)	260 (15.2)
**Ever smoked**, n (%)	152 (59.6)		555 (79.3)	1082 (69.0)
**MS Characteristics**				
Age at MS onset, years, mean (SD)	31.4 (11.4)	33.2 (10.0)	33.3 (10.2)	33.0 (10.0)
Age at MS diagnosis, years, mean (SD)	36.2 (11.1)	-		
**Current course**, n (%)				
Relapsing remitting	184 (72.2)	687 (72.4)	444 (62.7)	
Secondary progressive	48 (18.8)	193 (20.3)	116 (16.4)	
Primary progressive	23 (9.0)	60 (6.3)	67 (9.5)	
Possible/uncertain	0 (0)	0 (0)	81 (11.4)	
EDSS median (p25-p75)	4 (3-6)	2.5 (1.5-5.0)		

a- a- high school or less; b- any post-secondary education, remaining participants reported education as other;

EDSS = Expanded Disability Status Scale
