# Supplementary material for: Effects of Psychiatric Comorbidity in Immune-Mediated Inflammatory Disease: Protocol for a Prospective Study
Source: JMIR Res Protoc. 2018 Jan 17;7(1):e15. doi: 10.2196/resprot.8794 (PMC5792704; doi:10.2196/resprot.8794)
Supplement: Multimedia Appendix 3 [file resprot_v7i1e15_app3.pdf]

Multimedia Appendix 3. Characteristics of participants with rheumatoid arthritis (RA) and those of participants in other RA cohorts

**Characteristic**	**Present study**	**Siebert, Self-reported RA[93],[a]**	**Siebert, Treated RA[93],[a]**	**Dougados[94],[b]**	**Reed[95]**
Data collection years	2014-2016	2006-2010	2006-2010	2011-2012	2001-2014
Study name		United Kingdom Biobank	United Kingdom Biobank	COMORA	Corrona registry
Region	Manitoba, Canada	United Kingdom	United Kingdom	17 countries	United States
N	154	5657	2849	3920	29853
**Age, yr**					
Mean (SD)	59.5 (11.7)	-	-	56 (13) (48-63)	57.9 (13.4)
Median (p25-p75)		-	-		-
**Sex**, n (%)					
Male	24 (15.6)	1706 (30.2)	-	18.3% (9-33)	14660 (23.7)
Female	130 (84.4)	3951 (69.8)	-	81.7% (66-91)	15193 (76.3)
**Ethnicity**, n (%)					
Caucasian	114 (74.5)	5327 (96.1)	2716 (97.0)	-	-
Other	39 (25.5)	214 (3.9)	84 (3.0)	-	-
Missing	1	116	49	-	
**Education**, n (%)					
<High school	14 (9.0)				

High School/ GED	37 (24.0)				
College	45 (29.2)				
Technical/Trade	19 (12.3)				
Bachelor degree	39 (25.3)			24.5% (5.3-75)	
**Marital Status**, n (%)					
Single/never married	20 (13.0)				
Married/common law	93 (60.4)			69.7% (50-86)	
Divorced/separated/widowed	41 (26.6)				
**Lipid/hypertensive treatment**, n (%)	61 (39.6)	4228 (75.2)	2103 (73.8)		
**Smoking status**, n (%)					
Current	22 (14.3)	711 (12.7)	326 (11.5)	13.2% (0.9-48)	
Past	73 (47.4)	2850 (50.8)	1493 (52.8)		
Never	59 (38.3)	2045 (36.5)	1008 (35.6)		
Missing	0	51	22		
**Overweight/obese**, n (%)	101 (65.5)		50.7% (0-69)[c]		
**Rheumatoid arthritis characteristics**					
Age at RA onset (self-reported), mean (SD)	39.5 (14.8)				
Age at RA diagnosis, mean (SD)	41.5 (15.0)				
Swollen joints, mean (SD)	1.3 (2.5)				4.5 (5.6)
Tender joints, mean (SD)	4.7 (6.9)				4.6 (6.2)

mHAQ, mean (SD)	0.52 (0.51)				
HAQ, mean (SD)				1.0 (0.7) (0.7-1.5)	
Disease-modifying antirheumatic drug use	135 (87.7)		2849 (100)	43% (last 5 years)	9411 (47.0)[d]

a- This study reported characteristics of all persons enrolled with self-reported RA, as well as the subcohort taking disease-modifying anti-rheumatic therapies; b- This study reported average percentages across all sites, and range of lowest and highest values in parentheses; c-highest frequency of overweight/obesity observed in the USA; d-history of biologic disease-modifying anti-rheumatic drug use mHAQ = modified Health Assessment Questionnaire, HAQ = Health Assessment Questionnaire
