# Supplementary material for: Effects of Psychiatric Comorbidity in Immune-Mediated Inflammatory Disease: Protocol for a Prospective Study
Source: JMIR Res Protoc. 2018 Jan 17;7(1):e15. doi: 10.2196/resprot.8794 (PMC5792704; doi:10.2196/resprot.8794)
Supplement: Multimedia Appendix 4 [file resprot_v7i1e15_app4.pdf]

Multimedia Appendix 4. Characteristics of participants with depression or anxiety disorder and those of participants in other depression/anxiety disorder cohorts

**Characteristic**	**Present study**	**2012 Canadian Community Health Survey: Mental Health**[a]
Data collection years	2014-2016	2012
Study name	-	CCHS-MH
Region	Manitoba, Canada	Canada
N	308	2812
**Age, yr**		
18-34	92 (29.9)	30.9%[b]
35-49	106 (34.4)	28.6%
50-64	100 (32.5)	28.6%
65-74	6 (1.9)	8.7%
≥75	4 (1.3)	3.2%
**Sex**, n (%)		
Male	72 (23.5)	37.0%
Female	235 (76.6)	63.0%
**Ethnicity**, n (%)		
Caucasian	245 (79.8)	85.0%
Other	62 (20.2)	15.0%
Missing	1	
**Education**, n (%)		
<High school	17 (5.5)	19.5%
High school/ GED	85 (27.6)	15.3%
>High school	206 (66.9)	65.1%

**Marital Status**, n (%)		
Single/never married	111 (36.0)	30.7%
Married/common law	134 (43.5)	40.8%
Divorced/separated/widowed	63 (20.5)	28.6%
**Smoking status**, n (%)		
Current	65 (21.1)	30.8%[c]
Past	88 (23.6)	38.6%
Never	153 (49.7)	30.6%
**Body mass index**, n (%)		
<20	21 (6.9)	6.2%
20- <25	73 (23.8)	33.5%
25- <30	82 (26.9)	31.2%
30 - <35	56 (18.4)	18.4%
≥35	73 (23.8)	10.8%
**Comorbid conditions, n (%)**		
Heart disease	13 (4.2)	5.7%
Diabetes	21 (6.8)	9.1%
Hypertension	62 (20.1)	18.7%
Lung disease	74 (24.0)	--[d]

a- Weighted proportions from the Statistics Canada public use microdata file (PUMF) based on n=2812 (weighted proportion = 9.5%) of respondents reporting a professional diagnosis of a mood or anxiety disorder; b-Age 15-34 rather than 18-34 due to the coding of age groups in the CCHS-MH public use datafile; c-Includes both current daily and occasional smokers; d- Not assessed in the CCHS-MH survey
